# Development of a dynamic risk system for predicting the risk of recurrence and progression in patients with non-muscle-invasive bladder cancer after thulium laser resection of bladder tumor or transurethral resection of bladder tumor followed by intravesical BCG instillation

**DOI:** 10.3389/fonc.2023.1133161

**Published:** 2023-07-05

**Authors:** Jian-Xuan Sun, Ye An, Meng-Yao Xu, Chen-Qian Liu, Jin-Zhou Xu, Qi-Dong Xia, Shao-Gang Wang

**Affiliations:** Department and Institute of Urology, Tongji Hospital, Tongji Medical College, Huazhong University of Science and Technology, Wuhan, China

**Keywords:** non-muscle invasive bladder cancer, thulium laser, en-bloc resection of bladder tumor, BCG immunotherapy, transurethral resection of bladder tumor

## Abstract

**Background:**

The high recurrence rate of non-muscle-invasive bladder cancer (NMIBC) after tumor resection brings huge physical and financial burdens for patients. Several predictive models that predict the recurrence of patients with NMIBC have drawbacks in clinical practice. With the rapid development of therapeutic methods, more factors should be taken into consideration when constructing predictive model.

**Methods:**

We retrospectively enrolled 90 patients who were diagnosed as intermediate- or high-risk NMIBC and received a Thulium laser resection of bladder tumor (TmLRBT) or transurethral resection of bladder tumor (TURBT) followed by BCG instillation. Univariate Cox regression analysis and multivariate Cox regression analysis were performed to screen out the independent prognostic factors of recurrence free survival (RFS). A nomogram and risk index were constructed using these prognostic factors.

**Results:**

In this study, 22 patients suffered recurrence; 37 patients (41%) received TmLRBT, and over 90% patients completed intravesical BCG instillation for one year. The univariate Cox regression showed that surgery (TURBT vs TmLRBT), previous bladder tumor, tumor number, pathological stage, post-operative catheterization and number of BCG therapy were associated with RFS. The multivariate Cox regression revealed that surgery (TURBT vs TmLRBT) (HR = 3.16, 95%CI [1.02 – 9.83]); previous bladder tumor (HR = 4.03, 95%CI [1.41 – 11.54]); number of BCG therapy (HR = 0.89, 95%CI [0.84 – 0.95]) were independent prognostic factors. A nomogram was constructed and exhibited excellent capability in predicting the RFS with an AUC of 0.789, 0.848, 0.806 at 6-, 12- and 24-months respectively and a c-index of 0.822. Also, the calibration curve and decision curve analysis were performed to verify the predictive efficacy. The risk index was derived from the nomogram and also exhibited favorable capability in predicting the progression free survival (PFS) of patients.

**Conclusions:**

Patients who received TmLRBT, without previous bladder tumor history and had more intravesical BCG instillations are likely to have better RFS. The nomogram and the risk index which were constructed to predict the RFS and PFS of patients may help urologists to make clinical decisions and aid in precision medicine.

## Introduction

Bladder cancer (BCa) is the tenth most common cancer and the second common urological malignancy worldwide. It is more common in men than in women with approximately 573,000 new cases and 213,000 cancer deaths in 2020 (1). According to the invasion depth of bladder wall, BCa can be divided to non-muscle-invasive bladder cancer (NMIBC) and muscle-invasive bladder cancer (MIBC). NMIBC represents about the 75% of newly diagnosed BCa.

For now, tumor resection followed by a scheduled intravesical instillation is the standard treatment for intermediate- and high-risk NMIBC (2, 3). But even for those who received bacillus Calmette-Guérin (BCG) instillation, there are still a non-negligible recurrence rate and progression rate. Once the NMIBC progresses to MIBC, patients may have to receive a radical cystectomy which will impact the survival condition of them severely. Therefore, predicting the recurrence or progression status of NMIBC at early time can bring huge clinical benefits. Several models have been constructed to predict the prognosis of BCa over the last decades (4–6). However, limited by the diversity of patients and with the development of new treatments for BCa, it is necessary to construct new predictive models.

Recent years, lasers including holmium YAG and thulium YAG have been used for surgeries in urology. More and more clinicians have considered Thulium laser resection of bladder tumor (TmLRBT) as a potentially useful alternative to conventional transurethral resection of bladder tumor (TURBT) with less short-term complications and good therapeutic effect (7, 8). Although several studies reported no statistical significance of recurrence between TmLRBT and TURBT groups, one study has demonstrated a lower recurrence rate of TmLRBT than TURBT (9). Therefore, in this study, we retrospectively screened out 90 patients who were diagnosed as intermediate- or high-risk NMIBC and received either TmLRBT or TURBT followed by intravesical BCG instillation. We evaluated the potential risk factors of recurrence and constructed a novel nomogram (including TmLRBT) to predict the risk of recurrence. Moreover, a risk index which was derived from the nomogram model was constructed, and showed excellent predictive efficacy in predicting the risk of progression.

## Materials and methods

The research was approved by the Ethics Committee of the Tongji Hospital, Tongji Medical College, Huazhong University of Science and Technology (Grant number: TJ-IRB20210106). This observational and retrospective study was approved for exempting of informed consent.

We retrospectively searched the database of our institution, and finally screened out 90 patients who were diagnosed as NMIBC and received a TmLRBT or TURBT from August 2018 to December 2019. The inclusion criteria were: (1) Diagnostic confirmation of NMIBC by pathology. (2) Received TmLRBT or conventional TURBT. (3) Confirmed as intermediate- or high-risk NMIBC by the EAU risk stratification (10). (4) Received a scheduled BCG intravesical instillation. (5) Having complete follow-up and clinical data. The exclusion criteria were: (1) Patients with any other cancers before or after bladder cancer diagnosis. (2) MIBC or metastatic bladder cancer. (3) Unable to accept the BCG instillation treatment due to intolerance or other reasons. (4) Lack of relevant clinical data.

Patients’ imaging including ultrasonography, cystoscopy and computerized tomography of urinary system (CTU) were performed before operation and confirmed the locations and characteristics of tumors. All patients received either TURBT or TmLRBT were aware of the advantages and shortcomings of these two surgical procedures. The surgical operations were performed by experienced urologists. All the surgeries were performed with standard procedures. TmLRBT was performed by an en-bloc resection using a continuous-flow resectoscope and thulium laser system (Raykeen, China) with a power set at 30 W. Firstly, a circular incision was performed using a thallium laser at about 5 millimeters away from the edge of the tumor. Secondly, surgeon deepened the incision to the deep muscular layer of the bladder below the tumor. Finally, a separation between the serosa and the muscular layer of the bladder was made to get a complete tumor specimen by laser. As for the conventional TURBT, all visible tumors or suspicious mucosal lesions were resected with a monopolar loop electrode. The resection was extended to the deep muscle layer of the bladder, a specimen of the detrusor was obtained by a lower power setting. All specimens were sent to the histopathology department for further assessments.

Once the gross hematuria occurred after operation, a 4 h bladder irrigation would be maintained until no signs of bleeding. The time for removing the catheter was according to the specific conditions of patients and ranged from 2 to 5 days. An intravesical 30 mg gemcitabine instillation was performed within 24 h after operation at the first time. The operation details and post-operative data were recorded and collected. All patients were diagnosed as intermediate- or high-risk NMIBC, and a standard intravesical BCG instillation was recommended. According to the instruction, two weeks after the surgery, patients were given weekly 2 g BCG instillation for 6 weeks, then biweekly for 6 weeks, and then monthly for 10 months. For those who were high-risk NMIBC, monthly instillation would be extended to 1 – 2 years. After surgery, patients were required to receive cystoscopy and ultrasonography every 3 months for surveillance. Additional telephone follow-up was performed every month. Once suspected lesions or visible recurrence appeared, TURBT and biopsy were performed. All recurrences were confirmed by histopathology, and progression was defined as the metastatic disease or development of muscle-invasive tumor.

The pre-operative information of included patients was also retrospectively searched and collected from the database of our institution. The following data were finally recorded: gender, age, surgery (TmLRBT or TURBT), previous bladder tumor history, tumor size, tumor location, tumor stage, tumor grade, risk of patient, operative time, obturator nerve reflex (ONR), perforation, hematuria, irrigation, catheterization, days of hospital stay and number of BCG treatment.

All statistical analyses were performed by R software vision 4.1.1. Continuous data with normal distribution (exhibited as mean ± standard deviation [SD]) were compared by Student’s t-test. Mann–Whitney U-test was used to detect the differences of continuous data with skewed distribution (exhibited as median, interquartile range [IQR]). Categorical variables (exhibited as percentage) were compared by the Chi-square test or Fisher’s exact test. We first used univariate Cox regression analysis to evaluate the prognostic values of variables we mentioned above. A variable with a P value less than 0.1 was applied to multivariate Cox regression analysis. Then, we constructed a nomogram based on those variables screened from the multivariate Cox regression analysis. Three methods including the decision curve analysis (DCA), the receiver operating characteristic (ROC) curve and the calibration curve were performed to assess the predictive efficacy of the nomogram (11). A risk index derived from the nomogram model is constructed and calculated according to the following formula:


$$\text{risk index}=\sum_{i=1}^{n}coef\left(i\right)\cdot expr\left(i\right)$$

In this formula, coef(i) represents the hazard ratio (HR) of the ith prognostic factor, and expr(i) represents the normalized score of the ith prognostic factor.

## Results

Ultimately, 90 patients were enrolled in our analyses, and the basic information of them were shown in **Table 1**. Among them, 22 patients suffered tumor recurrence, and we stratified patients into two groups according to the recurrence. No statistical significance was observed between two groups for most clinical characteristics (gender, age, tumor number, tumor size, tumor location). But we did find differences existing between two groups: the previous bladder tumor history (P=0.008) and tumor stage (P=0.037). It is worth noting that surgery (TURBT vs TmLRBT) between two groups have a critical P value (P=0.05). The perioperative information was also exhibited in **Table 1**.

**Table 1:** Basic characteristics of included patients.

	All patients (n = 90)	No recurrence (n = 68)	Recurrence (n = 22)	P value
**Follow-up (median [IQR])**	23 [17.25,26.00]			
**Gender, n (%)**				0.762^a^
Male	72 (80.0)	55 (80.9)	17 (77.3)	
Female	18 (20.0)	13 (19.1)	5 (22.7)	
**Age (median [IQR])**	63.50 [56.00,68.00]	63.00 [56.00,67.00]	66.00 [58.50,68.00]	0.253^b^
**Surgery, n (%)**				0.05^a^
TmLRBT	37 (41.1)	32 (47.1)	5 (22.7)	
TURBT	53 (58.9)	36 (52.9)	17 (77.3)	
**Previous Bladder Tumor, n (%)**				**0.008** ^a^
No	75 (83.3)	61 (89.7)	14 (63.6)	
Yes	15 (16.7)	7 (10.3)	8 (36.4)	
**Tumor Number (median [IQR])**	1.50 [1.00,2.75]	1.00 [1.00,2.25]	2.00 [1.00,2.75]	0.438^b^
**Tumor Multiplicity, n (%)**				0.462^a^
Single	45 (50.0)	36 (52.9)	9 (40.9)	
Multiple	45 (50.0)	32 (47.1)	13 (59.1)	
**Tumor Size (median [IQR])**	2.00 [1.20,2.50]	2.00 [1.50,2.50]	1.75 [1.05,2.00]	0.408^b^
**Location, n (%)**				1^a^
Other	27 (30.0)	21 (30.9)	6 (27.3)	
Lateral	63 (70.0)	47 (69.1)	16 (72.7)	
**Stage, n (%)**				**0.037** ^a^
Tis	5 (5.6)	3 (4.4)	2 (9.1)	
Ta	36 (40.0)	32 (47.1)	4 (18.2)	
T1	49 (54.4)	33 (48.5)	16 (72.7)	
**Grade, n (%)**				0.56^a^
PUNLMP	2 (2.2)	2 (2.9)	0 (0.0)	
Low	19 (21.1)	16 (23.5)	3 (13.6)	
High	69 (76.7)	50 (73.5)	19 (86.4)	
**Risk, n (%)**				0.22^a^
Intermediate	18 (20.0)	16 (23.5)	2 (9.1)	
High	72 (80.0)	52 (76.5)	20 (90.9)	
**Operative Time (median [IQR])**	27.50 [25.00,38.75]	25.00 [23.75,40.00]	30.00 [25.00,35.00]	0.327^b^
**ONR, n (%)**				0.399^a^
No	82 (91.1)	63 (92.6)	19 (86.4)	
Yes	8 (8.9)	5 (7.4)	3 (13.6)	
**Perforation, n (%)**				1^a^
No	87 (96.7)	66 (97.1)	21 (95.5)	
Yes	3 (3.3)	2 (2.9)	1 (4.5)	
**Hematuria, n (%)**				0.222^a^
No	48 (53.3)	39 (57.4)	9 (40.9)	
Yes	42 (46.7)	29 (42.6)	13 (59.1)	
**Irrigation (median [IQR])**	25.00 [5.00,30.00]	23.50 [5.00,30.00]	26.00 [10.25,34.00]	0.152^b^
**Catheterization (median [IQR])**	2.00 [2.00,3.00]	2.00 [2.00,3.00]	2.00 [2.00,3.00]	0.428^b^
**Hospital stay (median [IQR])**	3.00 [3.00,3.00]	3.00 [3.00,3.00]	3.00 [3.00,4.00]	0.288^b^
**Progression, n (%)**				**<0.001** ^a^
No	80 (88.9)	68 (100.0)	12 (54.5)	
Yes	10 (11.1)	0 (0.0)	17.50 [10.25,19.00]	
**Number of BCG treatment (median [IQR])**	19.00 [18.25,25.00]	21.00 [19.00,25.00]	17.50 [10.25,19.00]	**<0.001** ^b^

P-value less than 0.05 indicated statistically significant and highlighted in bold.

IQR, interquartile range; TmLRBT, Thulium laser resection of bladder tumor; TURBT, transurethral resection of bladder tumor; PUNLMP, papillary urothelial neoplasms of low malignant potential; ONR, obturator nerve reflex.

a Tested with Fisher’s Exact Test.

b Tested with Kruskal-Wallis H Test.

**Figure 1:**
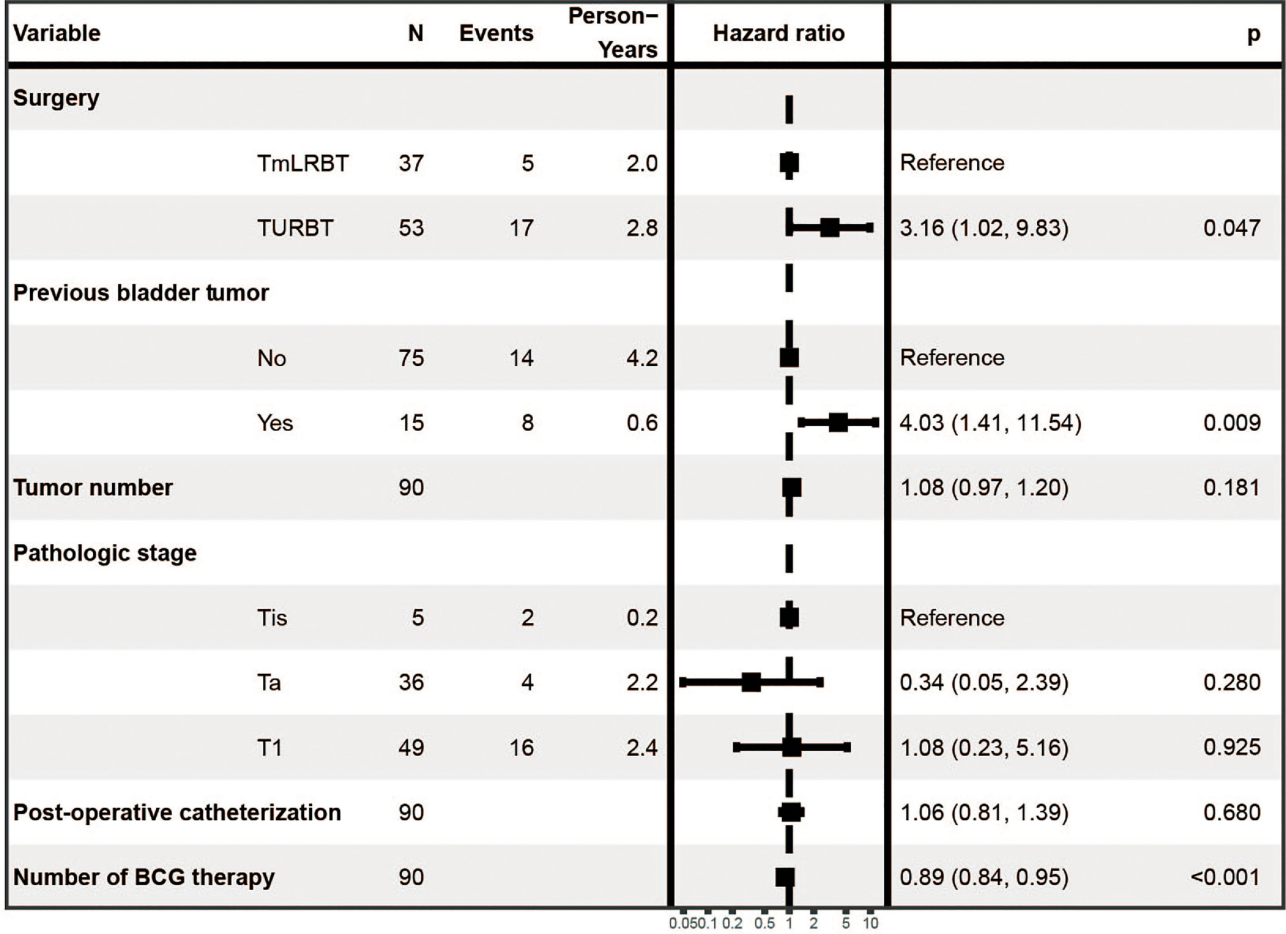
The multivariate Cox regression analysis.

We first performed univariate Cox regression analysis to screen prognostic factors for recurrence free survival (RFS) (**Table 2**), and set the statistical significance level as P< 0.1 for avoiding missing potential vital factors. Six variables including surgery (TURBT vs TmLRBT), previous bladder tumor, tumor number, pathological stage, post-operative catheterization and number of BCG therapy were screened out. The multivariate Cox regression revealed that three variables were independent prognostic factors of RFS: surgery (TURBT vs TmLRBT) (HR = 3.16, 95%CI [1.02 – 9.83]); previous bladder tumor (HR = 4.03, 95%CI [1.41 – 11.54]); number of BCG therapy (HR = 0.89, 95%CI [0.84 – 0.95]) (**Figure 1**).

**Table 2:** Univariate cox regression analysis of predictors of recurrence.

	HR	95%CI	P-value
**Gender (Female vs Male)**	1.21	0.44 - 3.27	0.713
**Age**	1.01	0.97 - 1.05	0.715
**Surgery (TURB vs TmLRBT)**	2.69	0.99 - 7.3	0.052
**Previous bladder tumor (Yes vs No)**	3.42	1.43 - 8.2	0.006
**Tumor Number**	1.1	1 - 1.21	0.06
**Tumor Multiplicity (Multiple vs Single)**	1.53	0.65 - 3.57	0.329
**Tumor Size**	0.81	0.5 - 1.31	0.387
**Location (Lateral vs Other)**	0.89	0.35 - 2.27	0.806
Stage			
Tis	–	–	Ref.
Ta	0.22	0.04 - 1.23	0.084
T1	0.76	0.18 - 3.34	0.721
Grade			
PUNLMP	–	–	Ref.
Low	187172.6	0 - Inf	0.998
High	0	0 - Inf	0.998
**Risk (High vs Intermediate)**	2.02	0.72 - 5.65	0.18
**Operative Time**	1.01	0.99 - 1.03	0.167
**ONR (Yes vs No)**	1.92	0.57 - 6.5	0.295
**Perforation (Yes vs No)**	1.43	0.19 - 10.65	0.726
**Hematuria (Yes vs No)**	1.8	0.77 - 4.21	0.176
**Irrigation (Yes vs No)**	2.31	0.78 - 6.82	0.13
**Irrigation**	1.02	0.99 - 1.06	0.206
**Catheterization**	1.23	0.98 - 1.56	0.079
**Hospital stay**	1.05	0.91 - 1.21	0.474
**Number of BCG treatment**	0.88	0.84 - 0.93	0

**Figure 2:**
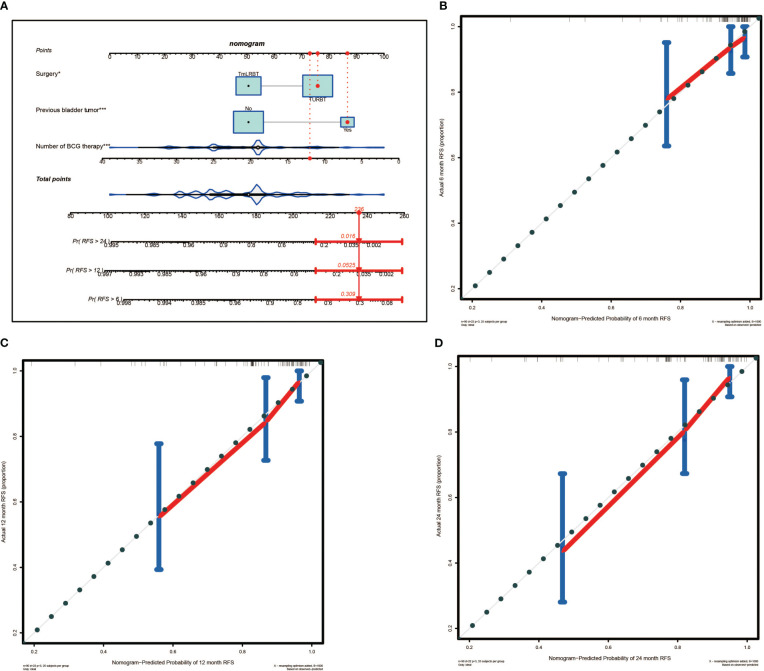
The nomogram for predicting the recurrence free survival (RFS) and the calibration curves. **(A)** Nomogram for patients predicting RFS. Surgery, previous bladder tumor and number of BCG therapy were included and marked as “points”. Total points by adding the three points can predict RFS. One patient who had a history of previous bladder tumor, and received a TURBT and 12 intravesical BCG instillations was randomly selected for analysis. By calculating, the predicted probability of RFS of 6-, 12- and 24-months are 0.309, 0.0525 and 0.016 respectively. The asterisks represented the statistical p value (*P< 0.05; ***P< 0.001). **(B)** The calibration curve of 6-months RFS. **(C)** The calibration curve of 12-months RFS. **(D)** The calibration curve of 24-months RFS.

**Figure 3:**
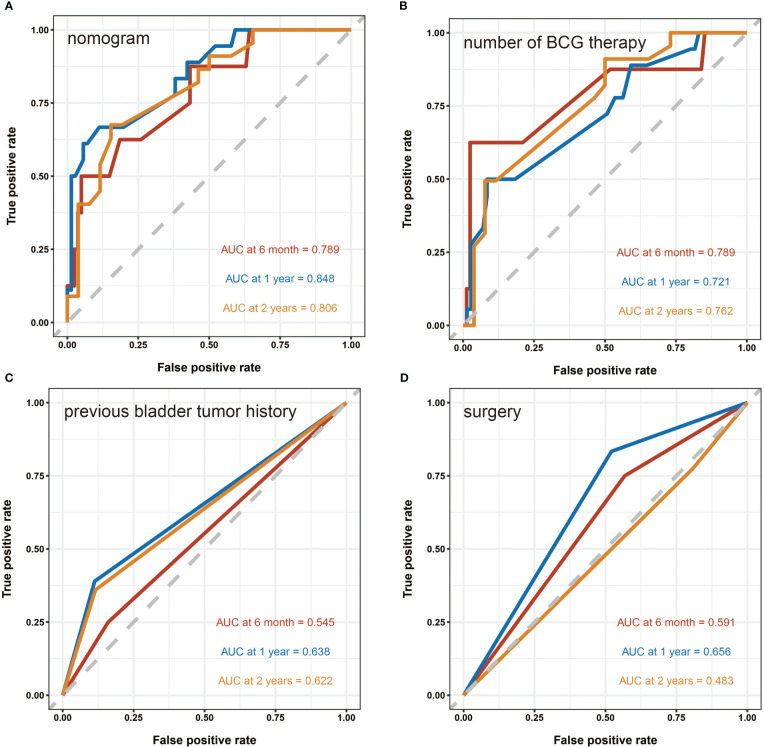
The receiver operating characteristic (ROC) curves. **(A)** The ROC curve of the nomogram. The area under curve (AUC) for the model at 6-, 12- and 24-months are 0.789, 0.848 and 0.806 respectively, which showed a favorable ability of predicting. **(B)** The ROC curve of the number of BCG therapy. **(C)** The ROC curve of the previous bladder tumor history. **(D)** The ROC curve of the surgery.

**Figure 4:**
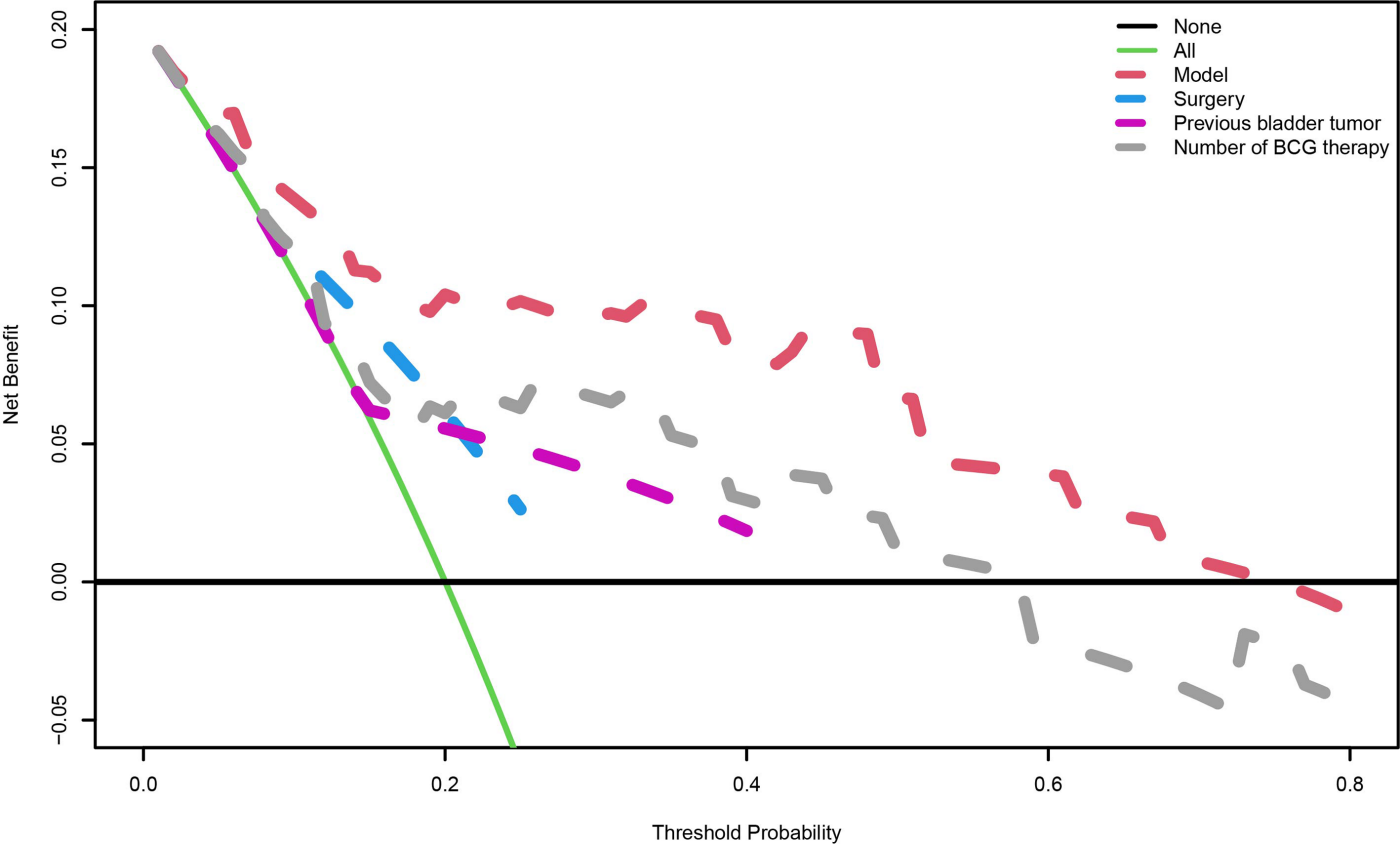
The decision curve analysis. When the risk threshold is around 0-73%, the net benefit of application of the model on taking measures is greater than the “treat-all-patient” or “treat-none” scheme. In addition, previous bladder tumor, number of BCG therapy or surgery alone is inferior to the model.

A nomogram predictive model was established based on the results of multivariate Cox regression to predict the RFS of patients (**Figure 2A**). Each status of these three variables was corresponding to a point, and after adding the three respective points, we would obtain a total point. The predicted RFS rate of 6-, 12- and 24-months were indicated by the axis under the total points axis. We randomly selected a patient who had a history of previous bladder tumor, and received a TURBT and 12 intravesical BCG instillations. After calculating, a total score of 236 was obtained, and the corresponding probability of RFS of 6-, 12- and 24-months were 0.309 (95%CI, [0.024 – 0.695]), 0.0525 (95%CI, [7.37e-05 – 0.402]) and 0.016 (95%CI, [1.6e-06 – 0.695]) respectively. The calibration curves of 6-, 12- and 24-months RFS were exhibited in **Figures 2B–D**, and we could observe an excellent predictive capability of the nomogram. The C index of our nomogram is 0.822 (95%CI, [0.778 – 0.866]). Furthermore, the ROC curve of the nomogram was plotted, and showed an area under curve (AUC) of 0.789, 0.848, 0.806 at 6-, 12- and 24-months respectively (**Figure 3A**), which was higher than the other independent predictive factors (number of BCG therapy, previous bladder tumor history and surgery) (**Figures 3B–D**). Finally, we performed DCA to further assess the predictive efficacy of the nomogram model (**Figure 4**). We found that our model always obtained the highest net benefit among “treat-none”, “treat-all-patients” schemes or other predictive factors irrespective of the threshold probability, which indicated a superior predictive efficacy.

**Figure 5:**
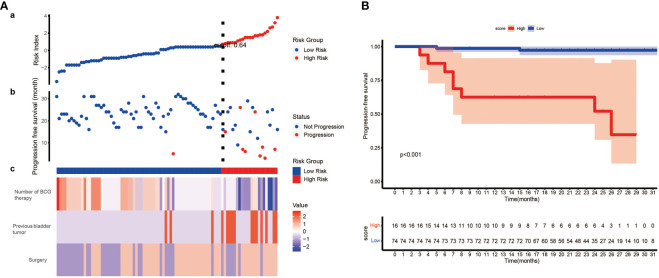
The development of risk index and its prognostic value. **(A)** (a) Risk index of each patient was calculated, and patients were stratified into high-risk index group and low-risk index group when setting 0.64 as the best cut-off value. (b) The progression status of each patient. (c) The normalized status of number of BCG therapy, previous bladder tumor and surgery of each patient. **(B)** Kaplan–Meier survival analysis indicates patients from low-risk group have significantly better PFS than those from high-risk group (P< 0.001, log-rank test). PFS, progression free survival.

To further explore the clinical value of our model, we constructed a scoring system named risk index. Using the formula we mentioned above, we calculated the risk index of each patient. Then, the best cut-off value (0.64) was identified, and patient who had a higher risk index than the cut-off value was regarded as high-risk and vice versa. By plotting the progression status of each patient, surprisingly, we noticed that patients in high-risk group were more likely to suffer tumor progression (**Figure 5A**). Therefore, we wonder whether the risk index could predict the progression free survival (PFS). The following survival analysis confirmed our hypothesis that patients from low-risk group had significantly better PFS than those from high-risk group (**Figure 5B**).

In general, we reported a prognostic value of TmLRBT compared to TURBT, and more importantly, we constructed a novel model involving the TmLRBT procedure to predict the recurrence or progression of patients with intermediate- or high-risk NMIBC.

## Discussion

Conventional TURBT, performed by an electrical wire loop, has been used and evaluated over decades, and is still the most commonly used surgery procedure for NMIBC. Nevertheless, there exist many drawbacks of TURBT, such as the thermal damage of specimens, unsatisfactory detection rate of detrusor muscle, tumor cells spreading and the risk of tumor cells seeding, which are considered to contribute to the recurrence or progression of NMIBC (12, 13). As an alternative to TURBT, the en-bloc technique requires a complete resection of tumor and catches more and more interest of urologists. Although it has potential to minimize the free tumor cells in bladder lumen and decrease the risk of tumor spreading, no obvious survival advantage of patients receiving en-bloc resection of bladder tumor (ERBT) was observed compared to conventional TURBT in prospective randomized trails (14, 15). However, it has been proved that the ERBT could decrease the presences of perioperative complications including bladder perforation and obturator nerve reflex (16). At the meantime, ERBT is capable of reducing the catheterization time and hospital stay time. It is worth to note that many studies set 3 cm as the cut-off value of tumor size in conducting ERBT, and Teoh et al. reported only a 29.6% success rate of ERBT when tumor size > 3 cm (17).

TmLRBT is one of the most commonly used procedures for ERBT. Comparing to other ERBT technologies such as Holmium YAG laser ERBT and kalium-titanyl-phosphate laser ERBT, TmLRBT shows several advantages: the lowest tissue penetration depth and a narrow incision line (13). That is the reason why we can observe an obvious trend using Thulium laser instead of Holmium laser. Besides, TmLRBT shows excellent hemostasis.

Although the consensus statement suggested it was safe to receive intravesical BCG instillation after ERBT (16), previous studies comparing the prognostic differences between TmLRBT and TURBT used chemotherapy drugs instead of BCG (18, 19). Therefore, in this study we included 90 patients who all received intravesical BCG instillations to investigate the prognosis differences between two procedures under BCG therapy, and over 90% patients received BCG treatment for one year. We first performed univariate and multivariate Cox regression analyses and identified 3 independent prognostic factors: surgery, previous bladder tumor history and number of BCG treatment. We found TmLRBT was correlated with better RFS than TURBT, which was inconsistent with previous studies. We proposed that this difference was caused by the usage of BCG. It is confirmed that tumor resection followed by BCG instillation was superior to tumor resection followed by chemotherapy instillation (20, 21). The BCG instillation might enhance the therapeutic efficacy of TmLRBT, and the combination of them synergically improve the RFS of NMIBC. Therefore, we supposed that it was of great benefit to receive a TmLRBT and a scheduled BCG instillation for those patients with intermediate- or high-risk NMIBC.

Notably, two famous predictive models, the EORTC scoring model and the CUETO scoring model, both include the prior recurrence status as prognostic factor for predicting the recurrence or progression of NMIBC (2, 4). We also found that the previous bladder tumor was an independent prognostic factor, and patients with previous bladder tumor history were associated with worse RFS than those who were not. Our results highlighted the clinical status of patients who suffered initial recurrence, and strict tumor surveillance and more clinical care are suggested for those patients. Shen et al. have reported the risk factors of repeated recurrence of patients suffered initial recurrence (22). In their study, time of instillation (docetaxel), tumor size, tumor grade, number of tumors, prior recurrence time were correlated with repeated recurrence.

Due to the toxicity of BCG, not all the patients could tolerant the whole schedule of BCG instillation. Therefore, different attempts have been carried out to reduce the toxicity, such as reducing the BCG dose of every instillation or reducing the frequency of instillations. A clinical trial (NCT00002990) has elucidated that no differences in toxicity existed between reduced BCG dose (1/3) and full BCG dose, but there did exist differences in cancer control that full dose with 3 year instillation significantly reduced the recurrence of high-risk NMIBC rather than reduced dose (23). It is widely accepted that BCG played its role through immune response (24), and the animal studies have proved that fewer number of BCG instillations were adequate to induce the immune response (25). However, a clinical trial has demonstrated that when considering the first time to recurrence, the reduced frequency of BCG instillations was inferior to standard frequency (26). In our study, we found more instillations of BCG was associated with better RFS. There results suggested that the standard dose and frequency (number) of BCG instillation was still the optimal choice for patients with intermediate- and high- risk NMIBC if they could tolerant.

Using these three prognostic factors, we constructed a nomogram. The calibration curves revealed that the model-predicted survival was highly consistent with the actual situation. The AUC of our model is significantly higher than other models, which exhibited an excellent predictive capability of the nomogram. The DCA curve, which is applied to investigate the clinical benefit when clinicians make clinical decisions using the predict models, showed that our model could instruct clinical decisions well, and obtained the best clinical benefits than treat none, treat all patients or other models (27). Finally, based on our model, we constructed a scoring system called risk index. Surprisingly, we found we could predict the PFS of patients by risk index, and patients from low-risk index group were correlated with significantly better PFS than those from high-risk group.

However, there are limitations in this study. First, the number of samples is limited, and this is the main drawback of our study. Second, the retrospective nature may lead to some inaccuracies of our results. Namely, the patients we included were all received intravesical BCG instillations, which led to a higher proportion of patients with high pathological grades. Third, as we mentioned above, TmLRBT is more likely to be performed when handling tumors with small size. Although the tumor size was not an independent prognostic factor in our study, this might still bring potential bias. Forth, until recent years, BCG treatment has been widely used for bladder cancer, and for those patients who suffered previous bladder tumor, most of them had received maintaining chemotherapy instillation such as epirubicin or gemcitabine. Besides, all simples came from a single institution and lacked external verification.

## Conclusion

A novel nomogram was established to predict the RFS, and patients who received TmLRBT, without previous bladder tumor history and had more BCG instillations are likely to have better RFS. Moreover, a risk index derived from the nomogram model showed excellent capability to predict the PFS of patients.

## Data availability statement

The original contributions presented in the study are included in the article/supplementary material. Further inquiries can be directed to the corresponding authors.

## Ethics statement

The studies involving human participants were reviewed and approved by Ethics Committee of the Tongji Hospital, Tongji Medical College, Huazhong University of Science and Technology (Grant number: TJ-IRB20210106). Written informed consent for participation was not required for this study in accordance with the national legislation and the institutional requirements.

## Author contributions

Q-DX and S-GW designed the study. Q-DX and J-XS collected the data, analyzed the data, and J-XS drew the figures. YA and J-XS wrote the manuscript. M-YX, C-QL and J-ZX contributed to critical revision of the manuscript. All authors contributed to the article and approved the submitted version.
